# Meta‐analysis of the role of entomopathogenic and unspecialized fungal endophytes as plant bodyguards

**DOI:** 10.1111/nph.15859

**Published:** 2019-05-13

**Authors:** Alan C. Gange, Julia Koricheva, Amanda F. Currie, Lara R. Jaber, Stefan Vidal

**Affiliations:** ^1^ School of Biological Sciences Royal Holloway University of London Egham Surrey TW20 0EX UK; ^2^ Department of Plant Protection School of Agriculture The University of Jordan Amman 11942 Jordan; ^3^ Department of Crop Protection Agricultural Entomology Georg‐August University Goettingen Grisebachstrasse 6 Goettingen 37077 Germany

**Keywords:** defence, effect size, entomopathogen, inoculation, insect herbivore, seeds, systemic growth

## Abstract

- Herbaceous plants harbour species‐rich communities of asymptomatic endophytic fungi. Although some of these endophytes are entomopathogenic, many are not, and remarkably little is known about how the presence of these fungi in plant tissues affects phytophagous insects.
- Here we show through a meta‐analysis that both entomopathogenic and nonentomopathogenic endophytes have a negative effect on insect herbivores. Growth and performance of polyphagous and sucking insects are reduced by nonentomopathogenic endophytes, but monophages are unaffected, likely because the latter are better adapted to secondary metabolites produced or induced by the fungi.
- Furthermore, studies using excised leaves report weaker effects than those with intact plants, likely caused by chemical changes being masked by leaf excision. Most surprisingly, endophyte infection of seeds produces the greatest effect on insect herbivores in subsequent mature plants, even though the usual mode of fungal transmission is infection of leaves by airborne spores.
- We conclude that these ubiquitous hidden fungi may be important bodyguards of plants. However, in order to fully understand their roles in plant protection, we must be aware that minor differences in experimental design can lead to contradictory results.

## Introduction

Every living plant contains endophytic fungi, which reside within the host tissues while causing no symptoms of disease (Bamisile *et al*., 2018). In grasses, Clavicipitaceous fungi often are relatively narrow in their host range and exhibit vertical transmission, via seeds. Due to enhanced production of secondary metabolites in colonized tissues, these fungi are often mutualistic and may protect plants against vertebrate and invertebrate herbivores. Given the agronomic importance of this group of endophytes, they have received much attention (Saikkonen *et al*., 2010; Liebe & White, 2018).

However, herbaceous plants contain a wide variety of unspecialized fungal endophytes, comprising latent pathogens, pathogens of other hosts, general saprotrophs and entomopathogenic species (Currie *et al*., 2014). These fungi exhibit most of the attributes of ‘Class III’ endophytes (Rodriguez *et al*., 2009), which are characterized by showing high diversity, having a broad host range, colonizing foliar tissues, having limited growth within the plant and mainly being transmitted horizontally from one plant to another, via airborne spores.

The latter two points have led some authors to speculate that these fungi should have very loose relationships with their host, being opportunistic colonizers that reside within plant tissues for limited periods of time (Boyle *et al*., 2001). Indeed, it has been suggested that such fungi do not engage in the mutualistic relationships with plants as seen in grasses, and thus will have no role as moderators of attack rates by herbivores and plant pathogens (Faeth, 2002). Given that a large proportion of any flora consists of nonwoody plants (FitzJohn *et al*., 2014) and that every plant suffers from insect herbivory, it is remarkable that this assertion has never been tested at a broad scale.

There are good reasons as to why these fungi may have significant interactions with insect herbivores on a given plant. First, many endophyte species produce an array of metabolites within plants, many of which may have anti‐herbivore activity (Nisa *et al*., 2015). Secondly, some of these fungi can be vertically transmitted from the parent plant to seedlings, via the seed, suggesting some form of mutualistic relation between host and endophyte (Hodgson *et al*., 2014; Quesada‐Moraga *et al*., 2014). However, in previous reviews, the inconsistency of effects of fungal presence on insect herbivores has been noted (Jaber & Vidal, 2010; Suryanarayanan, 2013), with variability in environmental and experimental conditions between studies suggested as a reason. This variability has constrained our understanding of endophyte–plant–insect interactions in natural communities and has limited their use in agriculture (Le Cocq *et al*., 2017).

Perhaps the most interesting group of Class III endophytes are the entomopathogenic fungi, some of which have been shown to colonize plant tissues asymptomatically, offering novel forms of biological pest control (Vidal & Jaber, 2015; Jaber & Ownley, 2018). Despite the pathogenicity of these fungi to insects, through both direct (infection and toxin production) and indirect (metabolite production in plants) pathways (Gurulingappa *et al*., 2011), previous studies also reported inconsistent effects, most likely caused by differences in experimental conditions (Vidal & Jaber, 2015). In order to exploit these fungi in crop protection and to understand their role in natural communities, there is a clear need for an overarching meta‐analysis that seeks to identify the general patterns of interactions between endophytes of herbaceous plants and insect herbivores and explores the causes of variation in effects. Similar meta‐analyses have been performed recently with plant pathogenic fungi (Fernandez‐Conradi *et al*., 2018), wherein biotrophic fungal presence was shown to reduce insect performance, perhaps mediated by crosstalk between plant defensive pathways. The above meta‐analysis also included ‘endophytes’, but Clavicipitaceous, entomopathogenic and nonentomopathogenic fungi were considered as one group, masking many important differences between them (Fernandez‐Conradi *et al*., 2018).

Inappropriate grouping together of fungal types has recently been shown to present considerable problems in the interpretation of meta studies involving mycorrhizal fungi (Brundrett & Tedersoo, 2019). Such errors include mis‐diagnosis of the fungi and poor or dubious recording techniques. The endophyte literature appears to suffer from similar problems (Currie *et al*., 2014), and we hypothesized that the variation in outcomes seen in studies of entomopathogenic and nonentomopathogenic fungi were caused by inappropriate methods or techniques. We therefore sought explicitly to address the influence of these problems in our study.

Here we use a dataset consisting of 527 case studies (effect sizes) to examine how unspecialized endophytes may affect the performance of herbivorous insects, and how experimental design may affect the outcome of the study. The entomopathogenic species are well described by Vega (2018); all belong to the fungal order Hypocreales and clearly have a very different biology to the other endophytes studied (in the orders Capnodiales, Eurotiales, Glomerellales, Mortierellales, Pleosporales, Polyporales, Sebacinales, Sordariales and Trichosphaeriales), hence we conducted our analyses separately for these entomopathogenic and nonentomopathogenic fungi. Previous meta‐analyses with plant pathogens (Fernandez‐Conradi *et al*., 2018) and arbuscular mycorrhizas (Koricheva *et al*., 2009) indicated that insect feeding mode is an important aspect to consider, as chewing and sucking insects often respond differently to plant defences. In addition, experiments involving endophytes use different modes of fungal inoculation and different ways of presenting leaf material to the insects, and hence we also investigated whether experimental conditions could explain the inconsistency of effects noted previously. Our results show that these hidden fungi have negative effects on the growth and performance of insect herbivores, but that sucking insects are more strongly affected. Polyphages and monophages respond differently, strongly suggestive of a chemical changes in the plant, caused by fungal presence. However, these important findings can be compromised by the design of any experiment.

## Materials and Methods

### Literature search

We searched the Web of Science (ISI) electronic bibliographic database (using the ‘All databases’ selection) for the period 1950–2018. We used a combination of search terms including insect* AND endophyt*, herbivor* AND endophyt*, entomopathogen* AND endophyt*, and all combinations of endophyt* with the eight insect orders that contain herbivorous species (Coleoptera, Diptera, Hemiptera, Hymenoptera, Lepidoptera, Orthoptera, Phasmida and Thysanoptera). We also used terms that identified different feeding modes: chewing, sucking, mining and galling, and diet breadth: polyphagous, oligophagous and monophagous, combining each with endophyt*. We identified important review papers (Rodriguez *et al*., 2009; Currie *et al*., 2014; Vidal & Jaber, 2015; Bamisile *et al*., 2018; Fernandez‐Conradi *et al*., 2018; Vega, 2018) and checked the references listed within each and citations of these. Finally, we supplemented the published literature with unpublished work, from theses produced in our laboratories. Use of such literature is recommended, as meta analyses based purely on published results can be biased, because significant results are more likely to be published (the so‐called ‘file drawer problem’) (Koricheva *et al*., 2013).

### Data collection and collation

Our initial search produced a total of 3595 experiments, spread across 1590 publications. We first removed the very few manipulative studies which pertained to woody hosts, and then those with Clavicipitaceous fungi in grasses, those which had used artificial diet, and those in which the endophyte species was not identified, enabling us to focus on foliar‐feeding insects and endophytes in herbaceous plants only. This left 665 separate studies spread over 67 publications. We then removed those studies where there were too few (< 5 as a conservative measure) observations of a response parameter to provide an acceptable sample. We also removed one experiment that contained the only five studies to use Orthoptera, as these were not independent. Initially, we identified 25 different insect parameters that had been recorded. Out of the following list, those in italics were used in the final analysis, having sample sizes > 5: *host choice*, birth weight, development rate, *development time*, approximate digestibility of food (AD), efficiency of conversion of digested food (ECD), efficiency of conversion of ingested food (ECI), *amount eaten*, feeding time, intrinsic rate of increase (Rm), *larval survival*,* larval weight*, pre‐pupal time, relative consumption rate, relative growth rate (MRGR), pupal period, pupal survival, *pupal weight*, adult deformity, adult longevity, adult survival, adult weight, *fecundity*, egg viability and *abundance (population count)*.

Most studies involved several parameters being measured, for example development time, larval weight and adult fecundity. We treated these as separate observations, because previous meta‐analyses showed that it is unusual for these separate life‐history parameters to all respond in the same way (Koricheva *et al*., 2009). Nevertheless, multiple outcomes from one study can be correlated (Koricheva *et al*., 2013) and so we also ran separate analyses based on a reduced dataset using randomly selected single independent recordings from each study. Some studies reported repeated observations (e.g. population counts) through time. In this case, we used data from the final recording of the experiment, to avoid temporal pseudoreplication. Some studies reported survival of insects, whereas others reported mortality rates. The latter were re‐calculated as survival rates, to avoid a potential contradiction of effects (e.g. increase in survival being a positive effect on insect, but increase in mortality a negative one). We also calculated the reciprocal of development time, so that increase in development time (detrimental effect on insects) resulted in decrease of the effect size. Having trimmed the dataset to remove these potential sources of bias, we ended up with 527 studies, spread over 54 publications. The publications are listed in Supporting Information Table S1. In addition to the response parameters listed above, we identified additional moderators (explanatory variables) of insect order, extent of diet specialization (monophagous, oligophagous or polyphagous, determined by literature searches for known hosts of each insect), insect feeding guild, plant family, whether the study was conducted using excised leaves fed to insects or on intact plants, and the mode of inoculation of the fungus (onto seeds, roots or shoots).

For each study, we extracted the sample size, mean value of the response variable and a measure of variation (standard deviation or standard error) for both inoculation treatment and control. When required, we extracted data digitally from graphics using image j software v.1.48 (Schneider *et al*., 2012).

### Statistical analysis

Analyses were conducted using the ‘OpenMEE’ interface (Wallace *et al*., 2017) of R/metafor v.3.4.1. Standardized mean difference (Hedges’ *d*) was used as a measure of the effect size to estimate the difference between insect herbivore performance on endophyte‐colonized and control plants. We then estimated the grand mean effect size for the whole dataset. However, it was clear that entomopathogens and other unspecialized endophytes differ greatly in their effects (using meta‐regression; (see below) (*Q*_m_ = 106.53, df = 1, *P *<* *0.001), and indeed are taxonomically different (entomopathogens are all in the order Hypocreales). We thus conducted subgroup meta‐analyses separately for these two broad groups of fungi. We considered a mean effect size to be significant when its 95% confidence intervals did not overlap zero, and in this study, a negative effect size means that a particular insect parameter is decreased by endophyte presence. We used a random‐effects model to combine effect sizes, due to considerable variation across studies. We then performed meta‐regressions to examine the different effects of moderator variables on insect performance and used the Restricted Maximum‐Likelihood method to estimate between‐study variance (Koricheva *et al*., 2013).

The effects of moderators were tested hierarchically, to avoid any potential nonindependence between them. Mixed effects models were used to estimate the effect of each moderator (insect order, degree of diet breadth, feeding guild, plant family, excised leaves/intact plants and method of inoculation) on the magnitude of insect performance. Such models make the assumption that the differences between studies within groups are due to random variation, whereas the variation between groups is fixed. We used the between‐group heterogeneity (*Q*_m_) to test for the significance of each moderator. We took significance of *Q*_m_ to show that the mean effect size differed between levels of the moderator, and further assumed that groups within the same moderator differed if their confidence intervals (CIs) did not overlap (Koricheva *et al*., 2013).

We also tested for bias in our dataset using three different approaches. First, we tested whether publication status of the study affected the magnitude of the effect, by comparing the results of published and unpublished studies in the different fungal parts of the dataset. There was no difference between these for entomopathogens (*d* (published) = −1.323, CI = (−1.471, −1.174), *n* = 294; *d* (unpublished) = −1.105, CI = (−1.469, −0.74), *n* = 28; *Q*_m_ = 0.663, df = 1, *P *>* *0.05) or nonentomopathogens (*d* (published) = −0.189, CI = (−0.353, −0.024), *n* = 196; *d* (unpublished) = 0.0402, CI = (−0.443, 0.527), *n* = 9; *Q*_m_ = 0.179, df = 1, *P *>* *0.05).

We also tested for publication bias by examining funnel plots (Figs S1, S2) and using the regression test for funnel plot asymmetry in ‘metafor’. We found no evidence of asymmetry for nonentomopathogens (*z *=* *−0.824, *P *>* *0.05) but we did for entomopathogens (*z *=* *−12.93, *P *<* *0.001). The latter plot is skewed by the nature of the data; most of the effect sizes are negative, and small standard errors occur when the outcomes are strongly negative.

Finally, we calculated the number of nonsignificant unpublished studies that would need to be added to make the results of the analysis nonsignificant, using Rosenberg's fail‐safe number. For nonentomopathogens, this was 1561, which is larger than 5N + 10 (1035) and for entomopathogens it was 84 534, again larger than 5N + 10 (1620). This indicates that our results are robust against possible publication bias.

## Results

Overall, in the full dataset, the mean effect size was strong and negative (*d *=* *−0.847, CI = (−0.960, −0.733), *P *<* *0.001, *n *=* *527), indicating that endophytic fungal presence significantly reduces insect growth and performance. To further test whether inclusion of multiple observations per study affected the results, we ran the model again on a reduced dataset that contained only single, randomly selected independent measurements from each experiment. This reduced dataset produced even stronger mean effect size (*d *=* *−1.008, CI = (−1.138, −0.879), *n *=* *418). As there was little difference in the mean effect size from these two analyses, and the CIs overlapped, we performed all subsequent analyses on the full dataset.

Entomopathogenic fungi occurring as endophytes had a strong negative effect on insect herbivores (*d *=* *−1.297, CI = (−1.436, −1.159), *n *=* *322). However, critically, the overall effect of nonentomopathogenic fungi on insects also was significantly negative (*d *=* *−0.181, CI = (−0.34, −0.023), *P *<* *0.05, *n *=* *205), but much weaker than that of entomopathogenic fungi (*Q*_m_ = 106.53, df = 1, *P *<* *0.001).

**Figure 1:**
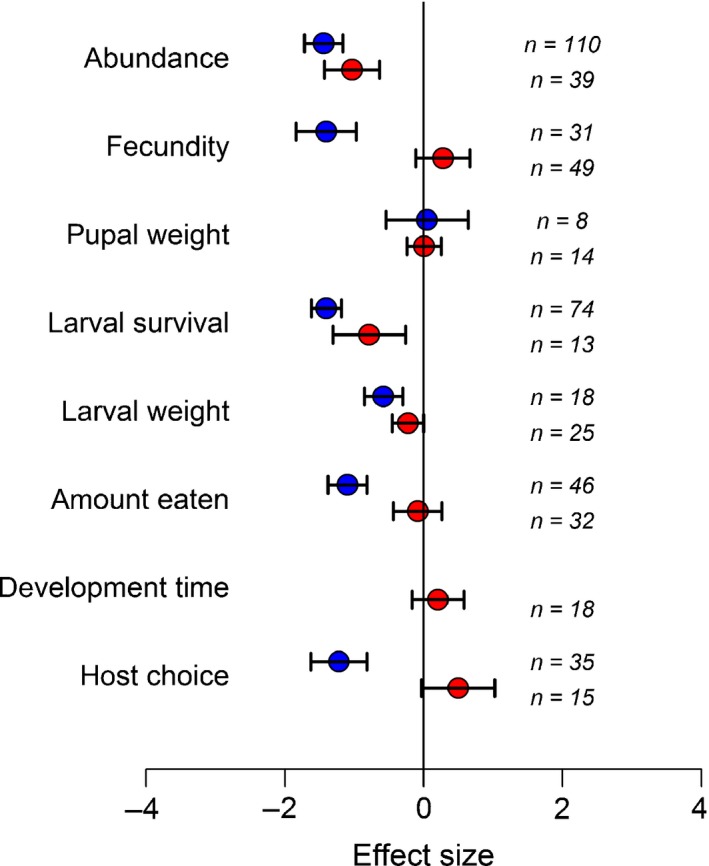
Entomopathogenic fungi (blue symbols) reduced all insect parameters, with the exception of pupal weight. Unspecialized, nonentomopathogenic fungi (red symbols) reduced larval weight and survival and thereby populations (abundance). Values are mean effect size (Hedges’ *d*) and negative values mean that the presence of the fungi reduces the insect performance parameter. Error bars represent 95% confidence intervals, and effect size is considered significant when these do not overlap zero (vertical line); *n*, number of effect sizes for each group.

Entomopathogens negatively affected all insect performance and preference parameters apart from pupal weight (Fig. 1), whereas nonentomopathogens significantly reduced larval survival and insect abundance (population counts), and had a weak (*P *=* *0.048) detrimental effect on larval weight. Also of interest is the parameter of host choice. Presence of entomopathogenic endophytes in plant tissues significantly reduced the preference of insects for colonized plants (Fig. 1), a feature that is likely to be of much interest in biological pest control. However, nonentomopathogenic fungal colonization of plant tissues did not repel insects; instead there was a weak evidence that these fungi may act as an attractant (*P *=* *0.065).

**Figure 2:**
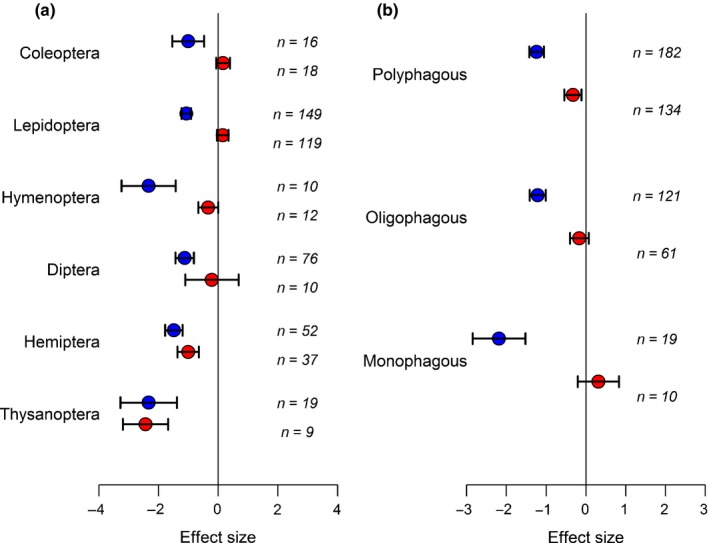
(a) Entomopathogens (blue symbols) were detrimental to all orders of insects, but nonentomopathogens (red symbols) only reduced the growth and performance of sucking insects. (b) The degree of feeding specialization is important, with unspecialized fungi being detrimental to polyphagous insects, but having no effect on specialists or those with a narrow diet. Values are mean effect size (Hedges’ *d*) and negative values mean that the presence of the fungi reduces insect performance. Error bars represent 95% confidence intervals, and effect size is considered significant when these do not overlap zero (vertical line); *n* denotes number of effect sizes for each group.

Entomopathogenic endophytes had detrimental effects on all insect orders, although sucking insects (Hemiptera and Thysanoptera) and Hymenoptera appear to be more affected (Fig. 2a). Intriguingly, the same three orders were the only ones affected by nonentomopathogenic fungal endophytes, although the effect on Hymenoptera was weak (*P *=* *0.048). The majority of studies on Hymenoptera involved host choice by polyphagous leaf‐cutting ants (*Atta* spp.) and the fact that the leaf material is not actually ingested by ants may explain why the effect of fungal presence was less pronounced. We included studies on leaf‐cutter ants in the analyses because these insects, although not herbivorous, are very important removers of plant tissues and can have devastating effects on the plants they attack. By contrast, some of the studies of entomopathogens and Hymenoptera involved galling insects, and such a sedentary lifestyle may render the insect a sitting target for the fungi, thereby producing a relatively large, negative effect. Six species of entomopathogens (*Beauveria bassiana, Hypocrea lixii, Metarhizium brunneum, M. anisopliae, Trichoderma asperellum* and *T. atroviride*) have been studied sufficiently to enable a comparison of their effects on insects. We found no evidence for differences between fungal species in the extent of antagonism (*Q*_m_ = 8.67, df = 5, *P *=* *0.123).

**Figure 3:**
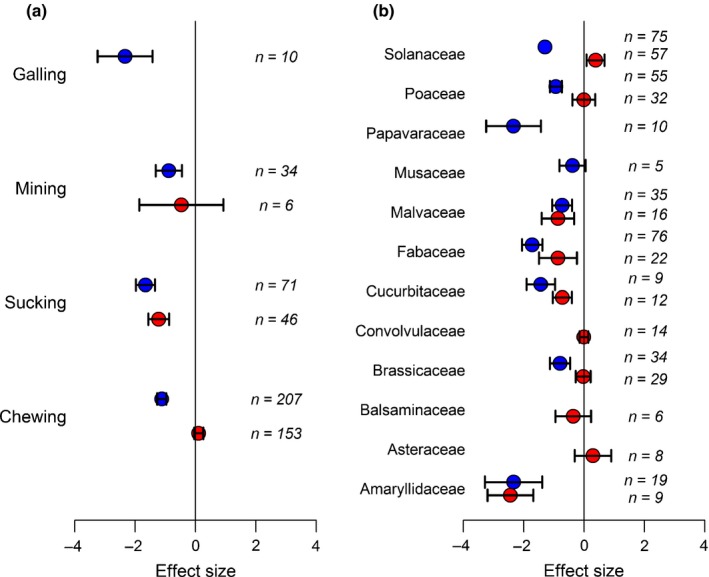
(a) Endophytic entomopathogens (blue symbols) reduced the growth of all insect feeding guilds, whereas effects of nonentomopathogens (red symbols) were seen only in sucking insects. (b) Effects of both entomopathogens and nonentomopathogens vary according to plant family. Values are mean effect size (Hedges’ *d*) and negative values mean that the presence of the fungi reduces insect performance. Error bars represent 95% confidence intervals, and effect size is considered significant when these do not overlap zero (vertical line); *n* denotes number of effect sizes for each group.

We found that insect responses to the presence of nonentomopathogenic endophytes depended on insect diet breadth (Fig. 2b), (*Q*_m_ = 9.66, df = 2, *P *<* *0.01), with polyphagous insects being the only group detrimentally affected by fungal presence (*d *=* *−0.328, CI = (−0.54, −0.116), *P *<* *0.05, *n *=* *134). This result contrasted with that for entomopathogens, in whose presence all categories of insects were detrimentally affected (*Q*_m_ = 2.08, df = 2, *P *=* *0.352) (Fig. 2b). The results for insect orders and diet were reflected in an analysis of feeding mode (Fig. 3a). With nonentomopathogenic endophytes, sucking insects were affected much more strongly by fungal presence (*Q*_m_ = 51.81, df = 2, *P *<* *0.001), whereas responses to entomopathogens also varied (*Q*_m_ = 19.76, df = 3, *P *<* *0.001). In the latter case, mining insects (in leaves and stems) were least affected.

There also were notable differences in the effects of the fungi within different plant families (Fig. 3b). Significant effects of nonentomopathogenic fungal presence on insect herbivores were observed only on host plants which belonged to the Amaryllidaceae, Cucurbitaceae, Fabaceae, Malvaceae and Solanaceae (*Q*_m_ = 68.76, df = 9, *P *<* *0.001). Entomopathogens had negative effects on insect herbivores associated with all plant families apart from Musaceae (*Q*_m_ = 42.77, df = 9, *P *<* *0.01), although here the sample size was very small (*n* = 5).

**Figure 4:**
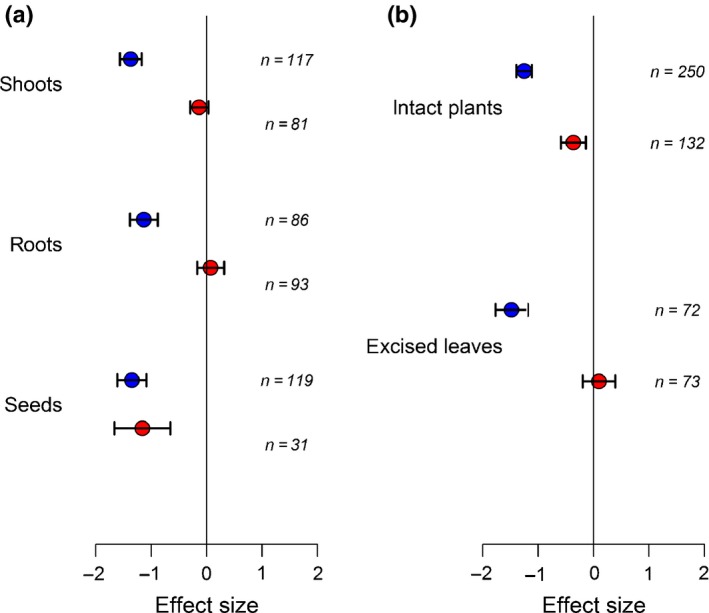
(a), Endophytic entomopathogens (blue symbols) reduced the growth of foliar‐feeding insects no matter what part of the plant they were applied to. However, inoculation of seeds with unspecialized endophytes (red symbols) was the only method that resulted in reduced insect growth. (b) Excision of leaves from plants colonized by unspecialized fungi seems to mask the effects seen when insects are reared on intact plants. Values are mean effect size (Hedges’ *d*) and negative values mean that the presence of the fungi reduces insect performance. Error bars represent 95% confidence intervals, and effect size is considered significant when these do not overlap zero (vertical line); *n* denotes number of effect sizes for each group.

The effects of entomopathogenic fungal endophytes on insects were detrimental irrespective of whether fungal inoculation occurred in roots, shoots or seeds (Fig. 4a). By contrast, it is remarkable that only seed inoculation with nonentomopathogenic fungi produced a detrimental effect on insects (Fig. 4a; *Q*_m_ = 25.15, df = 2, *P *<* *0.001). Thus, it is not surprising that variation in experimental outcomes has been noted before, because it would appear to be more intuitive to inoculate the leaves of a plant when conducting experiments with a horizontally transmitted fungus.

Further evidence for the influence of experimental design was provided by an examination of the way in which the studies were conducted. For entomopathogens, roughly one quarter, and for nonentomopathogens, roughly one‐third of studies fed excised leaves from colonized and control plants to insects, whereas the rest of the studies used intact plants. For entomopathogenic endophytes (Fig. 4b), use of excised leaves made no difference, but for nonentomopathogens, it determined the outcome of the experiment. Insects fed excised leaves showed no response to fungal presence, whereas those reared on intact plants, often with the same fungal species involved, were affected detrimentally (*Q*_m_ = 8.44, df = 1, *P *<* *0.01).

## Discussion

Our analyses have shown that unspecialized fungal endophytes in herbaceous plants reduce insect growth and performance. Importantly, both entomopathogens and nonentomopathogenic fungi reduce many insect performance parameters, but with the latter group the effect varies between insect orders and with the degree of insect host specialization. Moreover, for the first time, we showed that the fungal inoculation method and conduct of the experiment are vital in determining the outcome.

Strong negative effects of entomopathogenic fungi on insect herbivores revealed by our meta‐analysis are particularly remarkable considering that in all experiments these fungi were present as endophytes within the plant, and most insects were unlikely to have been in direct contact with the fungus. Indeed, in 93% of studies with endophytic entomopathogens, mycosis of the insect was absent (Vega, 2018). This result is important, as it suggests that there may be limited systemic growth of the fungi into the shoots and/or that acropetal, systemic chemical changes occur within the plant (Gibson *et al*., 2014; Jaber & Ownley, 2018). It is perhaps counter‐intuitive that greater effects were seen in sucking insects than in mining species, as one might expect the latter to have come into direct contact with the fungus as it grows. However, it has yet to be shown that fungal propagules produced endophytically can infect insects (Boomsma *et al*., 2014). Furthermore, studies reporting the exact location of the fungi within plant tissues are extremely rare. Entomopathogenic fungal growth may be intercellular, but limited (Ullrich *et al*., 2017), whereas growth within vascular bundles may be more extensive (Wagner & Lewis, 2000). Further studies are needed to determine if this is why sucking insects are more susceptible than internal miners or external chewers.

Limited fungal growth may explain why some of the studies in our analyses reported effects on insects while failing to recover the inoculated fungi. Nevertheless, the fact that entomopathogens can be inoculated on to seeds or roots and subsequently recovered from foliar tissues (Quesada‐Moraga *et al*., 2009; Akello & Sikora, 2012) shows that systemic acropetal growth must occur. Given that seed treatment can be as short as 2 h for infection to occur, and that plant growth enhancement can be seen in the absence of insects (Jaber & Enkerli, 2016), there is great promise for the exploitation of these fungi in seed dressings for agricultural use (Le Cocq *et al*., 2017).

Nonentomopathogenic fungal endophytes also have detrimental effects on insects, refuting the suggestion that they have no role in host protection (Faeth, 2002). Indeed, our results suggest that these fungi could be overlooked plant protection agents. Fungal presence seems to attract insects to plants, but this does not result in increased amounts of plant material consumed, in fact larval survival and thence population counts are reduced. However, it is intriguing that the only effects of these fungi on insects resulted from seed inoculation. These fungi have been recovered from seeds before, although germination represents a bottleneck for the fungus, leading to imperfect vertical transmission (Hodgson *et al*., 2014). Perhaps the critical difference is that all seed inoculation experiments soaked seeds in fungal suspensions for varying periods of time, hence the emerging seedling was likely to be in intimate contact with the fungal mycelium, in a humid environment. More difficult to explain is why foliar applications of endophytes had no effect, given that leaf infection by airborne spores is supposed to be the main method of fungal entry to aerial tissues (Rodriguez *et al*., 2009). We suggest that it may be a simple failure of technique, by not keeping leaf wetness sufficiently high (Dawson & Goldsmith, 2018). Plant pathologists have used dew chambers to obtain successful infections for over 40 yr (Clifford, 1973), and although some studies in our analysis attempted to raise humidity with polythene bags, none used a dew chamber. Thus, the way in which the experiment is conducted needs to be given considerable thought, because it can easily influence the outcome of the results.

Even if a seedling is colonized by a nonentomopathogenic endophyte, the fungus does not subsequently grow through the plant (Hodgson *et al*., 2014). A lack of systemic growth seems to characterize these fungi, and it is thought that recovery of a fungal species from different plant parts is due to separate spore infection events as the plant grows (Yan *et al*., 2015). However, infection by these fungi does result in novel and systemic chemical production within the host (Hartley *et al*., 2015), which is likely to begin at the seedling stage. Such chemical changes would explain why, like entomopathogens, effects on insects were reported without successful recovery of the fungus that was inoculated. Our study suggests that these chemical changes are more likely to be detrimental to polyphagous insects, which are less attuned to their host chemistry than are specialists (Jaenike, 1990). This situation is similar to that of root‐inhabiting arbuscular mycorrhizal fungi (AMF), wherein it has been shown that fungal presence also decreases the growth of generalist foliar chewing insects (Koricheva *et al*., 2009). The different effects on insects has led to the suggestion that AMF may have contributed to the evolution of insect specialism (Gange *et al*., 2002) because they also can increase the growth of sucking and specialist insects (Koricheva *et al*., 2009). We suggest that unspecialized endophytes also may have contributed to the evolution of insect specialism via chemical changes in plants resulting from fungal colonization. Furthermore, the endophyte community within forb species tends to be similar from one conspecific individual to another, but different between plant species growing in the same community (Gange *et al*., 2007). This may explain why we found a weak positive effect of these fungi on insect host choice, but also suggests that any plant species exerts a degree of control over the endophyte community within it. Taken together, these facts suggest that unspecialized endophytes should be included with entomopathogens as ‘plant bodyguards’ (Elliot *et al*., 2000), and supporting the suggestion that many of these saprophytic fungi have dual niches (Selosse *et al*., 2018).

The latter idea leads to the implication that entomopathogenic fungi could be considered as plant mutualists, in a similar manner to the closely related Clavicipitaceous endophytes (Barelli *et al*., 2016). This is supported by the recent discoveries that there is a bi‐directional transfer of nutrients between host plant and fungus, with insect‐derived nitrogen (N) passed to the plant and photosynthetically derived carbon (C) moving to the fungus (Behie *et al*., 2012, 2017). Whether such bi‐directional transfer occurs with other unspecialized endophytes is unknown, and this is clearly an avenue for future research. Intriguingly, it has recently been found that an unspecialized fungus, *Trimmatostroma* spp. does transfer N to plants when engaging in an ant–plant mutualism (Leroy *et al*., 2017). Meanwhile, although C may not be required from the host for fungal growth, it may well be required for metabolite production (Nisa *et al*., 2015).

Further evidence for the suggestion that endophyte‐mediated effects on insects stem from metabolite production is provided by the difference in results seen when insects are reared on intact plants or excised leaves. It has been known for over 30 yr that large chemical changes occur when a leaf is severed from a plant. Furthermore, altered nutrient availability in excised tissues also can mask any treatment effects and thereby influence the results of insect feeding tests (Risch, 1985). Thus, it seems remarkable that experiments continue to use such an artificial approach and we recommend that all future experiments use intact plants, to mimic the natural situation. Clearly the inconsistencies reported in the literature with nonentomopathogenic endophytes (Suryanarayanan, 2013) are due to inconsistent experimental design, as much as variation in environmental conditions. Furthermore, the fact that effects on insects differed between plant families (even when the endophytic fungus was the same) also strongly suggests that the mechanism is an indirect one, mediated through plant defences, rather than a direct effect of chemical production by the fungi.

Another design‐based problem of all the studies we examined is that the vast majority (98%) used single inoculations of fungi, often only quantifying recovery of the inoculated species, with no consideration of the background community (of either fungi or bacteria) within foliar tissues. Given that all herbaceous plants harbour a rich community of fungal endophytes (Currie *et al*., 2014) which varies from one plant species to another (Gange *et al*., 2007), this too could be a reason for previous inconsistency of effects. Indeed, in one of the few studies which used more than one fungal species, combinations produced different effects to single inoculations (Gange *et al*., 2012), perhaps explained by issues of functional complementarity between the endophyte taxa (Kia *et al*., 2017). Furthermore, the structure of a fungal endophyte community is affected by the presence of mycorrhizas in roots and interactions between the endophytes themselves (Gange *et al*., 2007; Eschen *et al*., 2010). Thus, we recommend that future studies consider using combinations of naturally co‐occurring endophytes, while also quantifying the background community of microbes. The interactions that the inoculated species have with this community might well be the source of metabolite production and thus effects on insects (Saunders *et al*., 2010).

The final deficiency in our knowledge highlighted by this study is the lack of consideration of higher trophic levels. In the few studies conducted, entomopathogenic fungal endophytes seem to have little or no effect on insect parasitoids, offering the opportunity for the use of both in field‐based pest control programmes (Gathage *et al*., 2016; González‐Mas *et al*., 2019). Meanwhile, beetle feeding on leaves of a tropical vine rich in endophytes increased the possibility of predation by ants nine‐fold (Hammer & Van Bael, 2015). This raises the intriguing possibility that unspecialized endophytes also could be determinants of insect community structure in natural situations. In the only experiment of its kind, application of *Trichoderma ghanense* and *T. harzianum*, which subsequently persisted as endophytes in a cabbage (*Brassica oleracea*) crop, resulted in changes in insect communities and increased rates of parasitism and predation (Zarate *et al*., 2015).

### Conclusions

We conclude that unspecialized fungal endophytes represent an important part of a plant's armoury against herbivorous insects and can be considered as bodyguards. These effects are particularly noticeable for insects feeding on phloem, in which many secondary metabolites are carried (Wink, 2010). Chemicals produced and/or induced (through signalling pathways) by both entomopathogenic and other unspecialized fungal endophytes and which travel systemically in plants, are likely to explain the effects seen in insects. However, experimental designs that compromise such chemical production and transport, such as leaf excision and failure to inoculate fungi in an appropriate manner, can bias the results. Future work needs to clarify the nature of the relationships between fungi and host, and to extend studies to include natural combinations of endophytes and the effects of their colonization on resident endophyte communities and higher trophic levels. In particular, the role that these hidden fungi play in determining populations and community structures of insects in natural situations needs to be addressed urgently.

## Author contributions

ACG and AFC developed the idea for this research; ACG and AFC extracted data and ACG conducted the analysis with JK; unpublished data were provided by SV; and ACG, JK, LRJ and SV wrote the paper and AFC contributed substantial revisions to the manuscript.

## Supporting information

Please note: Wiley Blackwell are not responsible for the content or functionality of any Supporting Information supplied by the authors. Any queries (other than missing material) should be directed to the *New Phytologist* Central Office.

**Fig. S1** Funnel plot test of asymmetry for nonentomopathogenic fungal endophyte data.
**Fig. S2** Funnel plot test of asymmetry for entomopathogenic fungal endophyte data.

**Table S1** List of studies used in the meta‐analysis.

## Data Availability

Data from this study are available via Figshare (doi: 10.17637/rh.8052881).
