# Supplementary material for: Meta‐analysis of the role of entomopathogenic and unspecialized fungal endophytes as plant bodyguards
Source: New Phytol. 2019 May 13;223(4):2002–10. doi: 10.1111/nph.15859 (PMC6766880; doi:10.1111/nph.15859)

Article acceptance date: 12 April 2019.

Supplementary figures associated with manuscript

Meta-analysis of the role of entomopathogenic and unspecialised fungal endophytes as plant bodyguards

Alan C. Gange, Julia Koricheva, Amanda F. Currie, Lara Jaber and Stefan Vidal

Tests of bias

Fig. S1 Funnel plot test of asymmetry for non-entomopathogenic fungal endophyte data.

There is no asymmetry in this plot

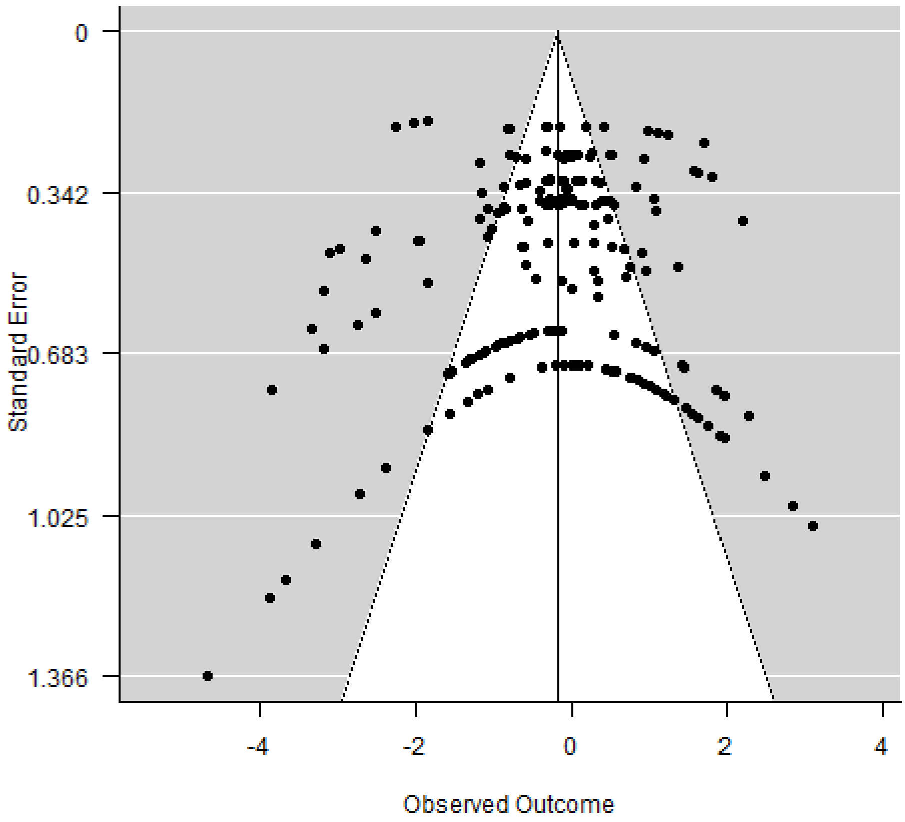

Fig. S2 Funnel plot test of asymmetry for entomopathogenic fungal endophyte data. There is significant asymmetry in this plot, caused by the fact that most effect sizes are negative, which is to be expected from insect-killing fungi.

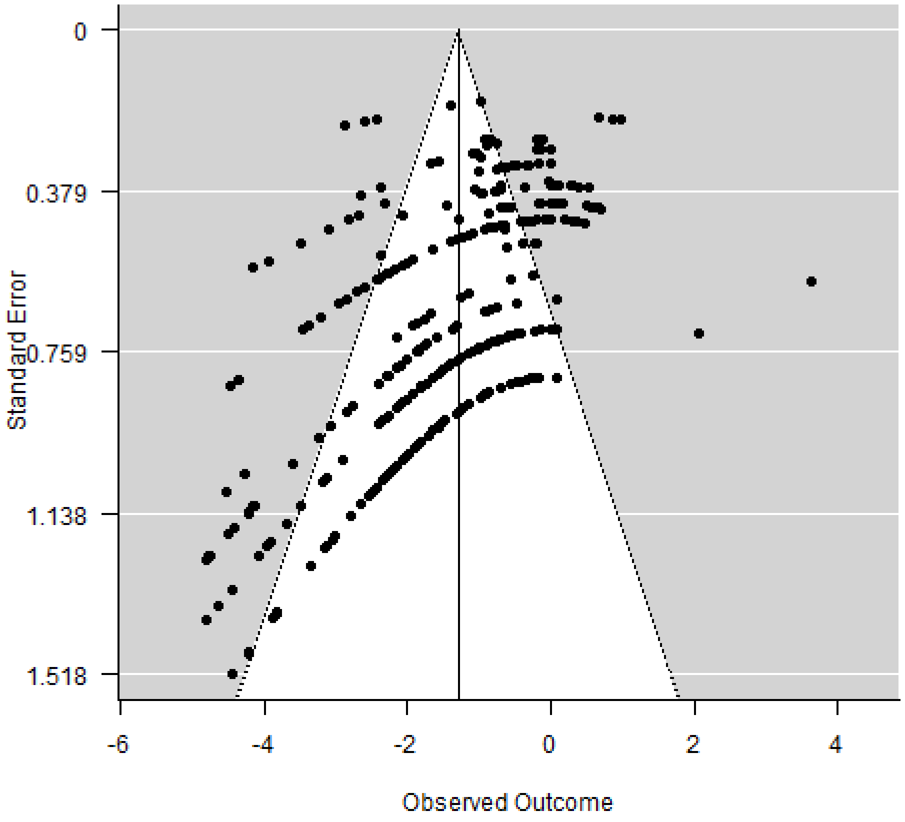

Supplement: Supplementary file 1 — Fig. S1 Funnel plot test of asymmetry for nonentomopathogenic fungal endophyte data. Fig. S2 Funnel plot test of asymmetry for entomopathogenic fungal endophyte data. [file NPH-223-2002-s001.pdf]
