# Supplementary material for: Meta‐analysis of the role of entomopathogenic and unspecialized fungal endophytes as plant bodyguards
Source: New Phytol. 2019 May 13;223(4):2002–10. doi: 10.1111/nph.15859 (PMC6766880; doi:10.1111/nph.15859)
Supplement: Supplementary file 2 — Table S1 List of studies used in the meta‐analysis. [file NPH-223-2002-s002.pdf]

Supporting Information Table S1: List of studies used in the meta-analysis, reported in

Meta-analysis of the role of entomopathogenic and unspecialised fungal endophytes as plant bodyguards

Alan C. Gange, Julia Koricheva, Amanda F. Currie, Lara R. Jaber and Stefan Vidal

Article acceptance date: 12 April 2019

In the table below, entomopathogenic fungal species are given in **bold type.**

Authors	date	title	publication	volume	pp	Fungi studied
Ab Razak, N.	2018	Integration of arbuscular mycorrhizal and endophyte fungi in the biological control of Himalayan balsam	PhD Thesis, Royal Holloway, University of London			Cladosporium sphaerospermum<br>Colletotrichum acutatum
Akello, J., Dubois, T., Coyne, D. & Kyamanywa, S.	2008	Effect of endophytic *Beauveria bassiana* on populations of the banana weevil, *Cosmopolites sordidus,* and their damage in tissue-cultured banana plants.	Ent. Exp. Appl.	29	157-65	**Beauveria bassiana**
Akello, J., Dubois, T., Coyne, D. & Kyamanywa, S.	2008	Endophytic *Beauveria bassiana* in banana (*Musa* spp.) reduces banana weevil (*Cosmopolites sordidus*) fitness and damage.	Crop. Prot.	27	1437-41	**Beauveria bassiana**
Akello, J. & Sikora, R.	2012	Systemic acropedal influence of endophyte seed treatment on *Acyrthosiphon pisum* and *Aphis fabae* offspring development and reproductive fitness	Biol. Cont.	61	215-21	**Beauveria bassiana**<br>Fusarium oxysporum<br>Gibberella moniliformis<br>**Hypocrea lixii**<br>**Metarhizium anisopliae**<br>**Trichoderma asperellum**

Akutse, K.S., Maniania, N.K., Fiaboe, K.K.M., Van den Berg, J. & Ekesi, S.	2013	Endophytic colonization of *Vicia faba* and *Phaseolus vulgaris* (Fabaceae) by fungal pathogens and their effects on the life-history parameters of *Liriomyza huidobrensis* (Diptera: Agromyzidae).	Fungal Ecol	6	293-301	**Beauveria bassiana**<br>Fusarium oxysporum<br>Gibberella moniliformis<br>**Hypocrea lixii**<br>**Trichoderma asperellum**
Alkhayat, D.	2016	Assessment of the risks and metabolic interactions in crops inoculated with entomopathogenic fungi	PhD Thesis, University of Göttingen			**Beauveria bassiana**
Aragon, S.	2016	How entomopathogenic endophytic fungi modulate plant-insect interactions	PhD Thesis, University of Göttingen			**Beauveria bassiana**<br>**Trichoderma koningiopsis**<br>**Metarhizium brunneum**
Barazani, O., Benderoth, M., Groten, K., Kuhlemeier, C. & Baldwin, I.T.	2005	*Piriformospora indica* and *Sebacina vermifera* increase growth performance at the expense of herbivore resistance in *Nicotiana attenuata*.	Oecologia	146	234-43	Sebacina vermifera
Batta, Y.A.	2013	Efficacy of endophytic and applied *Metarhizium anisopliae* (Metch.) Sorokin (Ascomycota: Hypocreales) against larvae of *Plutella xylostella* L. (Yponomeutidae: Lepidoptera) infesting *Brassica napus* plants	Crop. Prot.	44	128-34	**Metarhizium anisopliae**
Biswas, C., Dey, P., Satpathy, S., Satya, P. & Mahapatra, B.S.	2013	Endophytic colonization of white jute (*Corchorus capsularis*) plants by different *Beauveria bassiana* strains for managing stem weevil (*Apion corchori*).	Phytoparasitica	41	17-21	**Beauveria bassiana**
Castillo Lopez, D. & Sword, G.A.	2015	The endophytic fungal entomopathogens *Beauveria bassiana* and *Purpureocillium*	Biol. Cont.	89	53-60	**Beauveria bassiana**<br>**Phialemonium inflatum**

		lilacinum enhance the growth of cultivated cotton (*Gossypium hirsutum*) and negatively affect survival of the cotton bollworm (*Helicoverpa zea*).				
Castillo Lopez, D., Zhu-Salzman, K., Ek-Ramos, M.J. & Sword, G.A.	2014	The entomopathogenic fungal endophytes *Purpureocillium lilacinum* (formerly *Paecilomyces lilacinus*) and *Beauveria bassiana* negatively affect cotton aphid reproduction under both greenhouse and field conditions	PLOS One	9	e103891	**Beauveria bassiana** **Phialemonium inflatum**
Cherry, A.J., Banito, A., Djegui, D. & Lomer, C.	2004	Suppression of the stem-borer *Sesamia calamistis* (Lepidoptera; Noctuidae) in maize following seed dressing, topical application and stem injection with African isolates of *Beauveria bassiana*.	In. J. Pest Man.	50	67-73	**Beauveria bassiana**
Cherry, A.J., Lomer, C.J., Djegui, D. & Schulthess, F.	1999	Pathogen incidence and their potential as microbial control agents in IPM of maize stem borers in West Africa	Biocontrol	44	301-27	**Beauveria bassiana**
Cohen, S.	2017	The effects of Beauveria bassiana in Brassica oleracea var. italica on aphid performance.	MSc Thesis, Royal Holloway, University of London			**Beauveria bassiana**
Estrada, C., Degner, E.C., Rojas, E.I., Wcislo, W.T. & Van Bael, S.A.	2015	The role of endophyte diversity in protecting plants from defoliation by leaf-cutting ants.	Curr. Sci.	109	55-61	Colletotrichum tropicale

Estrada, C., Wcislo, W.T. & Van Bael, S.A.	2013	Symbiotic fungi alter plant chemistry that discourages leaf-cutting ants.	New Phytol.	198	241-51	Colletotrichum tropicale
Gan, H.J., Churchill, A.C.L. & Wickings, K.	2017	Invisible but consequential: root endophytic fungi have variable effects on belowground plant-insect interactions.	Ecosphere	8	e01710	**Beauveria bassiana**<br>Fusarium cf. equiseti<br>**Isaria fumosorosea**<br>Mortierella alpina<br>Trametes versicolor
Gange, A.C., Eschen, R., Wearn, J.A., Thawer, A. & Sutton, B.C.	2012	Differential effects of foliar endophytic fungi on insect herbivores attacking a herbaceous plant	Oecologia	168	1023-31	Alternaria alternata<br>Chaetomium cochliodes<br>Cladosporium cladosporioides<br>**Trichoderma viride**
Garrido-Jurado, I., Resquin-Romero, G., Amarilla, S.P., Rios-Moreno, A., Carrasco, L. & Quesada-Moraga, E	2017	Transient endophytic colonization of melon plants by entomopathogenic fungi after foliar application for the control of *Bemisia tabaci* Gennadius (Hemiptera: Aleyrodidae)	J. Pest Sci.	90	319-30	**Beauveria bassiana**<br>**Metarhizium brunneum**
Gathage, J.W., Lagat, Z.O., Fiaboe, K.K.M., Akutse, K.S., Ekesi, S. & Maniania, N.K.	2016	Prospects of fungal endophytes in the control of *Liriomyza* leafminer flies in common bean *Phaseolus vulgaris* under field conditions.	Biocontrol	61	741-53	**Beauveria bassiana**<br>**Hypocrea lixii**
Gurulingappa, P., Sword, G.A., Murdoch, G. & McGee, P.A.	2010	Colonization of crop plants by fungal entomopathogens and their effects on two insect pests when in planta.	Biol. Cont.	55	34-41	**Beauveria bassiana**<br>**Lecanicillium lecanii**
Hammer, T.J. & Van Bael, S.A.	2015	An endophyte-rich diet increases ant predation on a specialist herbivorous insect.	Ecol. Ent.	40	316-21	Colletotrichum tropicale
Hernawati, H., Wiyono, S. & Santoso, S.	2011	). Leaf endophytic fungi of chili (*Capsicum annuum*) and their role in	Biodiversitas	12	187-91	Aspergillus flavus<br>Coniothyrium spp<br>Nigrospora spp.

		the protection against *Aphis gossypii* (Homoptera: Aphididae).				
Jaber, L.R. & Vidal, S.	2009	Interactions between an endophytic fungus, aphids and extrafloral nectaries: do endophytes induce extrafloral-mediated defences in *Vicia faba*?	Func. Ecol.	23	707-14	Acremonium strictum
Jaber, L.R. & Vidal, S.	2010	Fungal endophyte negative effects on herbivory are enhanced on intact plants and maintained in a subsequent generation.	Ecol. Ent.	35	25-36	Acremonium strictum
Jallow, M.F.A., Dugassa-Gobena, D. & Vidal, S.	2004	Indirect interaction between an unspecialised endophytic fungus and a polyphagous moth.	Basic Appl. Ecol.	5	183-91	Acremonium strictum
Jallow, M.F.A., Dugassa-Gobena, D. & Vidal, S	2008	Jallow, M.F.A., Dugassa-Gobena, D. & Vidal, S.	Arthropod Pl. Int.	2	53-62	Acremonium strictum
Kaur, T., Singh, B., Kaur, A. & Kaur, S.	2015	Endophyte-mediated interactions between cauliflower, the herbivore *Spodoptera litura*, and the ectoparasitoid *Bracon hebetor*.	Oecologia	179	487-94	Aspergillus flavus<br>Aspergillus niger
Lewis, L.C., Bruck, D.J. & Gunnarson, R.D.	2002	On-farm evaluation of *Beauveria bassiana* for control of *Ostrinia nubilalis* in Iowa, USA.	Biocontrol	47	167-76	**Beauveria bassiana**
Li, L.	2009	Preference-performance relationships in herbivorous insects feeding on oilseed rape inoculated with soil-borne fungi.	PhD Thesis, University of Göttingen			Acremonium alternatum
Mahlanza, T., Rutherford, R.S., Snyman, S.J. & Watt, M.P.	2015	Potential of *Fusarium sacchari*-tolerant mutants in controlling *Eldana saccharina* and borer-associated *Fusarium* stem rot in sugarcane.	Eur. J. Plant Pathol.	141	825-37	Fusarium sacchari

Mantzoukas, S., Chondrogiannis, C. & Grammatikopoulos, G.	2015	Effects of three endophytic entomopathogens on sweet sorghum and on the larvae of the stalk borer *Sesamia nonagrioides*.	Ent. Exp. Appl.	154	78-87	**Beauveria bassiana**<br>**Metarhizium robertsii**<br>**Isaria fumosorosea**
Martinuz, A., Schouten, A., Menjivar, R.D. & Sikora, R.A.	2012	Effectiveness of systemic resistance toward *Aphis gossypii* (Hom., Aphididae) as induced by combined applications of the endophytes *Fusarium oxysporum* Fo162 and *Rhizobium etli* G12.	Biol. Cont.	62	206-12	Fusarium oxysporum
Menjivar, R.D., Cabrera, J.A., Kranz, J. & Sikora, R.A.	2012	Induction of metabolite organic compounds by mutualistic endophytic fungi to reduce the greenhouse whitefly *Trialeurodes vaporariorum* (Westwood) infection on tomato.	Plant & Soil	352	233-41	Fusarium oxysporum<br>**Trichoderma atroviride**
Mutune, B., Ekesi, S., Niassy, S., Matiru, V., Bii, C. & Maniania, N.K.	2016	Fungal endophytes as promising tools for the management of bean stem maggot *Ophiomyia phaseoli* on beans *Phaseolus vulgaris*.	J. Pest Sci.	89	993-1001	**Beauveria bassiana**<br>**Hypocrea lixii**<br>**Metarhizium anisopliae**<br>**Trichoderma asperellum**<br>**Trichoderma atroviride**
Muvea, A.M., Meyhofer, R., Maniania, N.K., Poehling, H.M., Ekesi, S. & Subramanian, S.	2015	Behavioral responses of *Thrips tabaci* Lindeman to endophyte-inoculated onion plants.	J. Pest Sci.	88	555-62	**Hypocrea lixii**
Muvea, A.M., Meyhofer, R., Subramanian, S., Poehling, H.M., Ekesi, S. & Maniania, N.K.	2014	Colonization of onions by endophytic fungi and their impacts on the biology of *Thrips tabaci*.	PLOS One	9	e108242	Clonostachys rosea<br>Fusarium sp.<br>**Hypocrea lixii**<br>**Trichoderma asperellum**<br>**Trichoderma atroviride**<br>**Trichoderma harzianum**

Qayyum, M.A., Wakil, W., Arif, M.J., Sahi, S.T. & Dunlap, C.A.	2015	Infection of *Helicoverpa armigera* by endophytic *Beauveria bassiana* colonizing tomato plants.	Biol. Cont.	90	200-7	**Beauveria bassiana**
Qi, G., Lan, N., Ma, X., Yu, Z. & Zhao, X.	2011	Controlling *Myzus persicae* with recombinant endophytic fungi *Chaetomium globosum* expressing *Pinellia ternata* agglutinin.	J. Appl. Microbiol.	110	1314-22	Chaetomium globosum
Quesada-Moraga, E., Munoz-Ledesma, F.J. & Santiago-Alvarez, C.	2009	Systemic protection of *Papaver somniferum* L. against *Iraella luteipes* (Hymenoptera: Cynipidae) by an endophytic strain of *Beauveria bassiana* (Ascomycota: Hypocreales).	Environ. Ent.	38	723-30	**Beauveria bassiana**
Raps, A. & Vidal, S.	1997	Development of two herbivores with different form of nutrition on endophyte-infected cabbage (*Brassica oleracea* var *gemmifera* L)	Mitt. Deutsch. Ges. All. Angew. Entomol.	11	1-6	Acremonium alternatum
Raps, A. & Vidal, S.	1998	Indirect effects of an unspecialized endophytic fungus on specialized plant-herbivorous insect interactions.	Oecologia	114	541-7	Acremonium alternatum
Razinger, J., Lutz, M., Schroers, H.J., Palmisano, M., Wohler, C., Urek, G. *et al.*	2014	Direct plantlet inoculation with soil or insect-associated fungi may control cabbage root fly maggots.	J. Invert. Pathol.	120	59-66	**Beauveria bassiana**<br>Clonostachys solani f. nigrovirens<br>**Metarhizium anisopliae**<br>**Metarhizium brunneum**<br>**Trichoderma atroviride**<br>**Trichoderma gamsii**<br>**Trichoderma koningiopsis**
Reddy, N.P., Khan, A.P.A., Devi, U.K., Shama, H.C. & Reineke, A.	2009	Treatment of millet crop plant (*Sorghum bicolor*) with the entomopathogenic fungus (*Beauveria bassiana*) to combat	J. Asia-Pacific Ent.	12	221-6	**Beauveria bassiana**

		infestation by the stem borer, *Chilo partellus* Swinhoe (Lepidoptera: Pyralidae).				
Resquin-Romero, G., Garrido-Jurado, I., Delso, C., Rios-Moreno, A. & Quesada-Moraga, E.	2016	Transient endophytic colonizations of plants improve the outcome of foliar applications of mycoinsecticides against chewing insects.	J. Invert. Pathol.	136	23-31	**Beauveria bassiana**<br>**Metarhizium brunneum**
Shrivastava, G., Ownley, B.H., Auge, R.M., Toler, H., Dee, M., Vu, A. *et al.*	2015	Colonization by arbuscular mycorrhizal and endophytic fungi enhanced terpene production in tomato plants and their defense against a herbivorous insect.	Symbiosis	65	65-74	**Beauveria bassiana**
Sword, G.A., Tessnow, A. & Ek-Ramos, M.J.	2017	Endophytic fungi alter sucking bug responses to cotton reproductive structures.	Insect Sci.	24	1003-14	**Beauveria bassiana**<br>**Phialemonium inflatum**
Thakur, A., Kaur, S., Kaur, A. & Singh, V.	2013	Enhanced resistance to *Spodoptera litura* in endophyte infected cauliflower plants.	Environ. Ent.	42	240-6	Cladosporium spp.<br>Nigrospora spp.
Van Bael, S.A., Seid, M.A. & Wcislo, W.T.	2012	Endophytic fungi increase the processing rate of leaves by leaf-cutting ants (*Atta*).	Ecol. Ent.	37	318-21	Colletotrichum tropicale
Van Bael, S.A., Valencia, M.C., Rojas, E.I., Gomez, N., Windsor, D.M. & Herre, E.A.	2009	Effects of foliar endophytic fungi on the preference and performance of the leaf beetle *Chelymorpha alternans* in Panama.	Biotropica	41	221-5	Glomerella cingulate
Vidal, S.	1996	Changes in suitability of tomato for whiteflies mediated by a non-pathogenic endophytc fungus.	Ent. Exp. Appl.	80	272-4	Acremonium strictum
Zhang, L.	2015	Colonization pattern of crop plants by endophytic fungi..	PhD Thesis, University of Göttingen			**Trichoderma harzianum**

Zhou, W.Q., Starr, J.L., Krumm, J.L. & Sword, G.A.	2016	The fungal endophyte *Chaetomium globosum* negatively affects both above- and belowground herbivores in cotton.	FEMS Microb. Ecol.	92	DOI: 110.1093/ femsec/fiw1158	Chaetomium globosum
